# Conducting Research with Tribal Communities: Sovereignty, Ethics, and Data-Sharing Issues

**DOI:** 10.1289/ehp.1103904

**Published:** 2011-09-02

**Authors:** Anna Harding, Barbara Harper, Dave Stone, Catherine O’Neill, Patricia Berger, Stuart Harris, Jamie Donatuto

**Affiliations:** 1 School of Biological and Population Health Sciences, College of Public Health and Human Sciences, Oregon State University, Corvallis, Oregon, USA; 2 Department of Science and Engineering, Confederated Tribes of the Umatilla Indian Reservation, Pendleton, Oregon, USA; 3 Department of Environmental and Molecular Toxicology, Oregon State University, Corvallis, Oregon, USA; 4 Seattle University School of Law, Seattle, Washington, USA; 5 Department of Information Technology, Marion County, Salem, Oregon, USA; 6 Swinomish Indian Tribal Community, Office of Planning and Community Development, La Conner, Washington, USA

**Keywords:** American Indian, data sharing, informed consent, intellectual property, IRB, research ethics, sovereignty, tribal

## Abstract

Background: When conducting research with American Indian tribes, informed consent beyond conventional institutional review board (IRB) review is needed because of the potential for adverse consequences at a community or governmental level that are unrecognized by academic researchers.

Objectives: In this article, we review sovereignty, research ethics, and data-sharing considerations when doing community-based participatory health–related or natural-resource–related research with American Indian nations and present a model material and data-sharing agreement that meets tribal and university requirements.

Discussion: Only tribal nations themselves can identify potential adverse outcomes, and they can do this only if they understand the assumptions and methods of the proposed research. Tribes must be truly equal partners in study design, data collection, interpretation, and publication. Advances in protection of intellectual property rights (IPR) are also applicable to IRB reviews, as are principles of sovereignty and indigenous rights, all of which affect data ownership and control.

Conclusions: Academic researchers engaged in tribal projects should become familiar with all three areas: sovereignty, ethics and informed consent, and IPR. We recommend developing an agreement with tribal partners that reflects both health-related IRB and natural-resource–related IPR considerations.

The value of community-based participatory research (CBPR) is well recognized [Israel et al. 2005; Minkler and Wallerstein 2008; National Institute of Environmental Health Sciences (NIEHS) 2010; U.S. Department of Health and Human Services (DHHS) 2010]. Successful CBPR requires a high level of collaboration to address community needs and to translate research findings to community members (Fleming et al. 2008; Glasgow and Emmons 2007; National Institutes of Health 2003). However, cultural differences, unrealistic expectations, organizational constraints, and ongoing ethical and data-sharing violations can create barriers that stifle or end effective partnerships between universities and sovereign tribal nations (Wong and Poon 2010). Few nonnative researchers possess an awareness of Native American culture and belief systems, including the continuing effect of American colonialism on the peoples they seek to study. For example, researchers may choose a model of “reductionist science” that relies on hypothetical quantitative models to determine disease excesses in communities with environmental contamination, while ignoring tribal community knowledge about health impacts and environmental abnormalities. Or, community members may not be included in scientific decision-making bodies that set standards of exposure and risk to community members (Quigley 2001). For university researchers unfamiliar with this history, it may be surprising when Native Americans are reluctant to engage in a proposed research project, even if the outcome is anticipated to be beneficial. Academics may also find it challenging to incorporate non-Western scientific paradigms within the constraints of a project. Or, tribal communities may insist on broadening the study scope to include urgent tribal health priorities or aims that were not within the initial project as funded and may not match narrow research goals of the funder.

Additional challenges may center on trust, data ownership, and sovereign rights. For example, there may be differences between conceptions of how knowledge may be generated, used, shared, and, ultimately, “owned.” Tsosie (2007) observed that in Western understandings, knowledge is generated by individuals who have autonomy in determining whether to share it. Once knowledge is shared, it is free for all to use, with only limited exceptions. By contrast, “within tribal communities, there may be an assumption that knowledge is part of the group’s overall identity, but that certain members have the duty to keep the knowledge on behalf of the group and that it would be inappropriate for such individuals to share the knowledge, even with other members of the group” (Tsosie 2007).

Past and ongoing abuses of tribal information underline the need for formalized data-sharing agreements specifically crafted for the tribal–university context. Some of these issues are considered at the institutional review board (IRB) level as they relate to basic human rights, informed consent, and beneficence. However, IRB approval from a university may not be sufficient to address tribes’ concerns because it may give the researcher free rein to acquire and publish tribal information. Moreover, IRB rules and requirements do not include a discussion of intellectual property rights (IPR). Likewise, IPR reviews do not include human subject principles such as beneficence, risks and benefits, or vulnerability. Neither the standard human subjects requirements nor IPR rules give adequate consideration to sovereignty or aboriginal rights, which is one of the reasons that inclusive declarations of indigenous rights have been published by the United Nations (2007) and are now recognized in principle by the world’s major powers, including the United States.

The generation of new and relevant data is a major goal of CBPR. Using CBPR methods, university and tribal scientists determine together the research aims and design, how data are collected, validated, and analyzed, and what results are needed to be useful both to the researchers and to the community. It is important to delineate early in the project how data will be handled beyond the traditional scope of university research rules and to resolve potential conflicts around data interpretation and publication (Resnick and Kennedy 2010). Factors that should be mutually agreed upon include the following:

Whether data include information heard in conversations, informal discussions, or social gatherings, and who is empowered to give associated permissions
Permissions and ownership of data collected formally or informally during the course of research
Protocols for the transport, storage, security, and retention of data
Principles of coauthorship and a transparent review process for publications, presentations, online postings, and other forms of information dissemination
Communication channels and timeliness of communication between stakeholders
Conditions for data analyses, including scope of research, privacy issues, and IPR.

We describe a university–tribal collaboration involving the Confederated Tribes of the Umatilla Indian Reservation (CTUIR) and Oregon State University (OSU) and present a model material and data-sharing agreement (MDSA). Although scientists at CTUIR and OSU acknowledge that an MDSA is only one element of successful CBPR with an American Indian nation, the agreement described here builds upon important ethical and legal principles and addresses challenges of trust, informed consent, data ownership, and sovereign rights. The premise of this article is not that information is unidirectionally disseminated, from university to tribe, but that the team of tribal and university researchers need to share data with each other and jointly work to provide information to various publics and governments.

## Rights of Sovereign Nations

American Indian tribes are sovereign governments. Tribes comprise distinct peoples, as that term is used in international law, with inherent rights to self-determination (United Nations 2007). Their status as self-governing sovereign entities predates contact with European settlers and was affirmed early on by the United States in *Worcester v. Georgia* (1832). Tribes continue to exist today as distinct sovereigns within the boundaries of the United States. This sovereign status is a defining feature of American Indian tribes, and it differentiates them from other “communities” with whom researchers might work (Quinault Indian Nation 1975).

As is true of all sovereign nations, tribal governments have a responsibility to ensure the well-being of their homeland and people, including cultural and intellectual patrimony. The preamble to the Constitution of the Quinault Indian Nation, for example, emphasizes preserving their land base, culture, and identity (Quinault Indian Nation 1975). Yet, tribes’ concerns as sovereigns can be understood only in light of their historical experiences. American Indian history is replete with efforts to terminate tribes as separate political entities and to assimilate tribal members into non-Indian society (Clinton et al. 2007). These frequently brutal efforts extend well into the memory of living tribal citizens and have profound and lasting effects. These efforts have denigrated the land-based facets of tribal cultures and denied the existence or value of traditional environmental knowledge.

Scholars and tribal leaders connect the continued sovereignty of tribes with the flourishing of tribal cultures. “Native peoples tend to see their cultures as encompassing systems of knowledge and understanding that are fundamental to the continuation of the tribe itself. Any harm to culture is perceived as a direct harm to the ability of the tribe to continue into the future” (Tsosie 2007). Knowledge itself, while held by individual custodians, may nonetheless belong to the tribe as a collective, that is, to be “part of the group’s overall identity” (Tsosie 2007). In addition, traditional environmental knowledge, stories, and other intellectual property are important sources of a tribe’s collective wealth and the inheritance of succeeding generations (Cruickshank 1990; Harris S, unpublished data; Tsosie 2002). The tribal governmental interest in safeguarding its cultural and intellectual patrimony is distinct from related interests that might be embraced by non-Indian governments within the United States.

Many tribes today, in an exercise of sovereignty, have set the terms under which research affecting their homeland, people, and culture will be conducted. For example, the Ho-Chunk Nation’s Tribal Research Code states that “the Legislature also has a fundamental responsibility to protect and preserve the culture of the Nation and to ensure that IRB permitted activities are conducted in a way that does no harm to the culture of the Nation” (Ho-Chunk Nation 2005). The code’s provisions apply to “all research conducted within the Nation’s Territory, whether involving human subjects or not, and all research regarding materials wherever located as to which the Nation has a claim of intellectual, cultural or other ownership, legal or equitable” (Ho-Chunk Nation 2005). Researchers must apply for and secure a permit, and tribal governments have the power to disapprove research proposals or to condition their approval. This is vastly different from conducting research in typical municipalities, where permission of the mayor or city council is not required.

Tribal governments are the only ones with authority to “speak for” the tribe as an entity. It is important for outsiders to recognize that each tribe and tribal community is unique. For example, tribal governments may be organized differently, tribal decisions may be made via differing formal and informal processes, and authority in various spheres (e.g., political, traditional, or cultural) may reside in different places (Cornell 1988; Williams 1994). Respectful researchers will need to work with the tribe to identify appropriate avenues, protocols, and experts relevant to their proposed work.

## IRBs, Codes of Ethics, and IPR Related to Tribal Research

Despite formal recognition of tribal sovereignty, indigenous communities and events are often not recognized as extensions of sovereign governments. Additionally, there is an entrenched Western mindset that indigenous worldviews of studying natural phenomena and human health, developed over centuries, are deficient and primitive (Beauchamp and Childress 1994; DeLoria et al. 1999; Jonsen et al. 1998). Differences between tribal and academic researchers are compounded when researchers are not present when needed, or make only a few visits, or simply leave upon completion of the project, as has often occurred in the past. Moreover, tribal needs may not accommodate research timelines, which are often too short (short-term or one-time federally funded initiatives) or too long (publication-heavy research without actual remedies for the community). It is rare that a federally funded initiative is timely and sustained, that the grant is received when needed by the tribe, and that the tribe is ready with adequate staff and processes in place.

American Indians’ circumstances present situations that require greater efforts at informed consent. Tribes are often more vulnerable because they are in the difficult position of seeking data and research funds while struggling against simply “being studied.” The inherent coercion must be minimized as a core tenet of bioethics (Jonsen et al. 1998). Furthermore, modern research may be so complex that even a fully competent nonspecialist might not understand the disclosed information accurately enough to make a truly informed decision (Minogue 1996). The ability of a tribe to give fully informed consent requires extra explanation and/or trained tribal staff who can consider the risks and benefits from a perspective inside the subject group’s legal, political, and cultural milieu. In addition, it is worthwhile to consider how the research will affect the tribal community as a whole, beyond the risks that may be incurred by the individual participants in the research project (Resnick and Sharp 2006). CBPR projects that help build capacity (skills, understanding, data, or equipment) within a tribe help overcome obstacles to informed consent.

Despite advances in general bioethics, federal initiatives such as environmental justice, and international recognition of indigenous rights (United Nations 2007), there are recent examples of missteps, such as the Havasupai case where members of the tribe accused researchers of improperly using tribe members’ blood samples in genetic research. This case resulted in significant adverse impacts to indigenous peoples [Mello and Wolf 2010; National Congress of American Indians (NCAI) 2006]. Equally disrespectful are the academic habits of attending tribal events, interviewing tribal members, and then writing first-author publications without IRB review or informed consent and tribal permission, or of failing to work with tribal researchers and then misinterpreting tribal information but publishing results as if they were accurate (Delistraty et al. 2010a, 2010b; Harris and Jim 2010). These academic practices irritate tribal scientists, perpetuate inaccuracies (Donatuto and Harper 2008), and do a great disservice to tribes and harm to tribal members because of publication of false information (Quigley 2001).

A positive example of collaboration is the Wabanaki Traditional Cultural Lifeways Exposure Scenario (Harper and Ranco 2009). In addition to the fundamental benefit of funding the tribes to develop their own report, this process represents an example of true intergovernmental consultation. Although the scenario development did not involve human subjects research, the consequences of underestimating environmental exposure rates could affect tribal health and sovereignty, so the principles of informed consent were followed. Because tribal leaders were not trained in risk assessment methodology, the approach and assumptions were discussed with tribal leaders and staff. Throughout the duration of the project, each tribe (through designated representatives) gained a basic understanding of the process, methods, and risks and benefits, while retaining control over the substance of the report.

## University–Tribal MDSA

To date, an appropriate MDSA between tribal communities and outside researchers has not been published in the literature. Such an agreement can provide and enforce equitable exchanges of information that benefit the community without infringing on the privacy of the study participants or on the sovereign rights of the tribe.

Table 1 summarizes current references to tribal codes of ethics related to research and IPR. The references cited in Table 1 formed the foundation for the initial MDSA of an NIEHS-funded university–tribal collaborative study between CTUIR and OSU [see Supplemental Material (http://dx.doi.org/10.1289/ehp.1103904)]. In addition to conventional items, the MDSA addresses data ownership and publication processes. This document incorporates input from the team of OSU and CTUIR researchers, the project’s Tribal Advisory Committee, OSU’s IRB and Research Contracts offices, the CTUIR Health Commission, and the Portland Area Indian Health Board. The MDSA was reviewed and approved by each organization’s legal officials. The final agreement has the following components:

**Table 1:** Codes of ethics and IPR and data-sharing agreements for tribal research.

Source	Title of document	Major points
Alaska Native Knowledge Network 2000		Guidelines for Respecting Cultural Knowledge		IPR and research rules (beneficence, protocol reviews); research involving Aboriginal Peoples; First Nations Code of Ethics pamphlet
American Anthropological Association 1998		Code of Ethics of the American Anthropological Association		Informed consent, working relationships, respect
American Indian Law Center, Inc. 1999		Model Tribal Research Code		Common pitfalls; Indian Health Service IRBs; research protocol review; benefits, rights, and enforcement
Australian Institute of Aboriginal and Torres Strait Islander Studies 1999		Guidelines for Ethical Research in Indigenous Studies		Principles of ethical research in Indigenous studies; practical applications, such as full and equal participation, affecting livelihoods, maintaining culture and heritage
Canadian Institutes for Health Research 2007		CIHR Guidelines for Health Research Involving Aboriginal People		Guidelines in conducting ethical and culturally competent research involving Aboriginal peoples
Desert Knowledge Cooperative Research Centre 2009		DKCRC Aboriginal Knowledge and Intellectual Property Protocol Community Guide		Intellectual property negotiation using Aboriginal paintings; ethics, confidentiality, free prior informed consent, benefit sharing, and research findings; reporting and publishing; protocols and research checklists
Freeman 2004		The Protection of Potential Individual Volunteers and Tribal Communities in Research Involving the Indian Health Service		Indian Health Service policies to protect participants and tribal communities; IRB composition; approval of research and publications by tribal communities; tribal informed consent
Hansen and VanFleet 2003		Traditional Knowledge and Intellectual Property: A Handbook on Issues and Options for Traditional Knowledge Holders in Protecting Their Intellectual Property and Maintaining Biological Diversity		Unpublished information and intellectual property of Tribes; patent process for native plants; publication ethics
Indian Health Service 2006; U.S. DHHS 2005		Human Research Participant Protection in the Indian Health Service		Indian Health Service IRB requirements; implications for 45 CFR 46 (Title 45, Code of Federal Regulations part 46), the Belmont Report, and the instructions of the Indian Health Service IRB(s) with jurisdiction
Indigenous Peoples Council on Biocolonialism 2000		Indigenous Research Protection Act		Legal protection for tribes in ownership of research; includes a model academic research agreement
International Society of Ethnobiology 2006		ISE Code of Ethics		Framework for decision making and conduct for ethnobiological research and related activities; code of ethics
Louis and Grossman 2009		Discussion Paper on Research and Indigenous Peoples, Association of American Geographers		Indigenous knowledge and intellectual property; revision of drafts and findings by tribes
Macaulay 1994		Ethics of research in Native communities		Benefits of the research to the community; ownership of data and publication issues
Macaulay 1998		Participatory research with Native community of Kahnawake creates innovative code of research ethics		Code of research ethics regarding ownership of data
Maddocks 1992		Ethics in Aboriginal research: a model for minorities or for all?		Ownership of data and publication; communication of results
National Science Foundation 2004a		Guidelines for Improved Cooperation between Arctic Researchers and Northern Communities		Guidelines for Arctic residents to shape research; consideration of subsistence lifestyles; includes checklist
National Science Foundation 2004b		Principles for the Conduct of Research in the Arctic		Principles for researchers in a broad spectrum of academic fields when conducting research in Arctic or northern regions
Navajo Nation 1996		Navajo Nation Human Research Code		Protections for Navajo people; conditions for physicians, researchers, and others doing research within the Navajo Nation
NCAI Policy Research Center 2010		Research That Benefits Native People: A Guide for Tribal Leaders		Five-module curriculum; research review policies and boards; selecting suitable research partners; applying values and ethics; understanding program evaluation
Quigley 2001		Compilation on environmental health: research ethics issues with Native communities		Partnership guidelines when working with indigenous communities
Sahota 2007		Research Regulation in American Indian/Alaska Native Communities: Policy and Practice Considerations		Ethical and legal considerations for American Indian/Alaska Native communities in regulation of research; American Indian/Alaska Native models of research ethics; legal justifications for American Indian/Alaska Native governments to regulate research
Sharp and Foster 2002		Community involvement in the ethical review of genetic research: lessons from American Indian and Alaska Native populations		Benefits and challenges of directly involving communities in the ethical review of research
World Health Organization 2010		Indigenous People and Participatory Health Research: Planning and Management, Preparing Research Agreements		Issues covered by a research agreement and examples of forms to be used with indigenous communities for an agreement, collective consent, informed consent

General project scope and collaborator: States the purpose of the project, the identity of the organizations participating in the agreement, the length of the agreement, procedures for its amendment or termination, and basic definitions.
Types of material and data collected: States the types of material and data to be collected and the general collection method. This includes data, such as analytical sampling results and demographic attributes, as well as collected organic material, transcripts of focus group discussions, and project-specific questionnaires.
Constraints on material and data use: Assures that materials and data supplied by the tribe to researchers or collected by researchers on behalf of the tribe are and remain tribal property and are not to be shared with third parties without the written permission of tribal authorities. It includes procedures for publication and postcompletion return of all materials and data.
Data access and security: Details the procedures for maintaining the physical security of the data, such as providing locked storage areas for paper documents and encrypting electronic media, and restricts data access to approved project researchers who require it for a specific task.
Risks and benefits of research to the tribal community: Summarizes the risks and benefits to be expected from participation in the research project, for both the individual and the tribal community.
Agreed-on mutual review processes: As a two-way document, the CTUIR agreed that it has equal responsibility for timely completion of research tasks and reports.

Developed in tandem with the MDSA were informed consent forms and confidentiality agreements. Although the focus of this article is on informed consent at the tribal government level, individual informed consent is equally imperative if the research involves human subjects. Whether researchers plan to attend a community gathering such as a pow wow and survey participants or to hold focus groups with tribal elders, individual informed consent is necessary for the same reasons as informed consent in a government context. Extra time and care must be taken to ensure that individuals know what they are consenting to, including clear and concise descriptions of the purpose of the research, use and storage of the information collected, and issues of anonymity. Moreover, many tribal organizations have their own IRBs or require researchers to obtain IRB approval from an organization such as the Indian Health Service, in addition to any academic IRB review. Tribally affiliated IRBs are necessary to ensure against potential adverse impacts to tribal individuals or governments that may be overlooked by academic IRBs and therefore are not redundant review processes.

The informed consent form provides potential tribal participants with straightforward information on the risks and rewards of project participation. The confidentiality agreements are required to be signed by university research personnel who have access to project material and data. These forms are held by the tribal researchers under secured conditions so that they know who has access to the data and for what purpose.

## Conclusion

The MDSA between CTUIR and OSU explicitly states agreed-on processes for the purposes of transparency for the benefit of university researchers and tribal governmental officials and other reviewers, and newer investigators and students. Mutually accommodating, the MDSA includes provisions such that both entities share equal responsibility for meeting project schedules and timely review of publications and grant reports. As tribes build scientific capacity, the collaboration model has moved from collecting data from tribes and reporting information back to them, to one of an equal tribal–university partnership in the research and in the dissemination of results to federal, community, and academic constituencies.

## Supplemental Material

(102 KB) PDF
