# Supplementary material for: Conducting Research with Tribal Communities: Sovereignty, Ethics, and Data-Sharing Issues
Source: Environ Health Perspect. 2011 Sep 2;120(1):6–10. doi: 10.1289/ehp.1103904 (PMC3261947; doi:10.1289/ehp.1103904)
Supplement: (102 KB) PDF [file ehp.1103904.s001.pdf]

Supplemental Material

Conducting Research with Tribal Communities: Sovereignty, Ethics and Data- Sharing Issues

Anna Harding, Barbara Harper, Dave Stone, Catherine O'Neill, Patricia Berger, Stuart Harris, Jamie Donatuto

Material and Data Sharing Agreement

CTUIR-OSU Collaboration to Address Tribal Exposures to PAHs and Improve Community Health

The parties to this agreement are The State of Oregon acting by and through the State Board of Higher Education on behalf of Oregon State University, an educational institution having offices at 312 Kerr Administration Building, Corvallis, Oregon 97331-2140 (“OSU”), Battelle Memorial Institute, Pacific Northwest Division, under its own right and under the authority of Contract No. DE-AC05-76RL0 1830 for the management and operation of the Pacific Northwest National Laboratory for the US Department of Energy, with offices at 902 Battelle Blvd., Richland, WA 99354 (“PNNL”), and the Confederated Tribes of the Umatilla Indian Reservation (“CTUIR”).

The purpose of this Agreement is to facilitate sharing of tribal related data, including public health data (both individually identified and population-related) as well as information about tribal practices, results from focus groups or individual members, and data collected on the Umatilla Indian Reservation, among the team members of the Community Outreach Core and Project 6-PAHs in Highly Exposed Populations of OSU’s NIEHS Superfund Research Center PNNL, the CTUIR tribal agencies, and tribal community members, to address the public health problems associated with polycyclic aromatic hydrocarbons (PAH) exposure on the Reservation and to assist in human capacity building with tribal partners. Any other member of the Superfund Research Center who handles tribal-related material or data is also included in this Agreement.

1. Period of Agreement

The period of this Agreement shall be in effect from 1/1/2010 until 3/31/2014, or until terminated in writing by either organization.

2. Definitions

i. Material means all particulate matter, urine samples, and preserved fish and game.

ii. Data means all information obtained from the CTUIR, tribal members, Material analysis, and results of Data processing.

3. Data to be collected

These projects shall collect Material and Data required to fulfill the following tasks:

A. Assessment of PAH exposure pathways specific to air quality and CTUIR traditional practices.

i. Ambient air monitoring stations will collect particulate matter associated with agricultural burning, coal-fired utilities, rural sources, and diesel emissions at the local truck stop. This information will be used to characterize the levels of PAH, nitro-PAH, and oxy-PAH of the general area.

ii. Personal air-monitoring equipment worn by volunteers will sample the level and type of PAHs present during food smoking activity in Tribal smoke sheds. Participants will also keep a diary noting the time and location of their activities. If participants agree to urine testing before and after food smoking activity, additional data collection will include measurements for chemical compounds that indicate the level of an individual’s

particulate-bound PAH exposure in urine. Participants will also be asked to fill out a brief survey to determine job position, verify non-smoking status, and other possible exposures to PAHs, such as smoked food, and secondhand smoke.

iii. Samples of preserved fish (salmon) and possibly, game will be analyzed to assess the distribution of PAH levels in food prepared by various indigenous preparation methods, including for example, pit cooking, smoking, and fresh caught. Material analysis will include PAH, oxy-PAH, and nitro-PAH measurements.

iv. Data collected from the air monitoring stations, from the personal air samplers, from urine samples, from the sampling of traditionally prepared foods, and from the written survey given to participants will be processed in Core C. Data processing conducted in Core C will compare/contrast the composition of the air masses obtained from Project 6 to those obtained from the air monitoring stations on the Reservation. Other analyses of interest will be the personal air exposures (from air and urine samples) obtained from Tribal members who are smoking foods, and analysis of PAH levels as described above in section Aiii. Data processing will also provide the project with the results of survey analysis to aid in determining exposure from smoking foods.

B. Development of culturally appropriate risk reduction approaches and outreach strategies that offer the best opportunity for improved health.

i. Discussions with CTUIR members will be held to help refine the Tribal Department of Science and Engineering (DOSE) working definition of "environmental health", which includes individual and community physical, mental, social, cultural, and spiritual well-being. Primary data output will be transcripts of discussion sessions.

ii. OSU and the DOSE will develop an overall picture of the existing general health conditions for the Tribe based on Indian Health Service summary health statistics. The outcomes will include appropriate public and medical health metrics related to overall cultural and community well-being.

iii. The design of risk reduction and health promotion strategies will be done in collaboration with various Tribal agencies, such as CTUIR nutrition staff, the Health Commission, or Yellowhawk Clinic. Data products will likely include health promotion approaches, mitigations strategies, and meeting transcripts.

iv. Dissemination of culturally appropriate risk reduction and health promotion information will require interactive sessions with interested Tribal departments, including the Tribal Health Commission, Yellowhawk Clinic medical staff, and the Tribal Board of Trustees. Through these meetings, OSU and tribal members will jointly develop culturally appropriate methods and strategies for information dissemination. Data outputs will include meeting transcripts and discussion documents.

4. Ownership and Constraints on Use of Material and Data

Material and Data supplied by CTUIR to OSU or to PNNL, or collected by OSU on behalf of CTUIR, is and remains the property of CTUIR and shall not be shared with third parties without the written permission of CTUIR. Participant data shall not be sold or used, internally or externally, for any purpose not directly related to the scope of work defined in this agreement without the written permission of CTUIR.

5. Data Access and Security

The identities of study participants will be kept confidential and all Material and Data will be coded and stored in secure storage areas (e.g., file cabinets or freezers), or on digital media that has been password protected, encrypted, or otherwise secured. Material and data will be de-identified prior to being shared between research projects, and will take place via a password-protected website hosted by PNNL that will provide investigators timely access to data while maintaining data confidentiality. Only members of research staffs who have signed the Confidentiality Agreement shall have access to the Material and Data. Prior to any Material or Data transfer under this Agreement, all staff members who will have access to the information will be notified of the use and disclosure requirements. Also prior to the transfer of any Material or Data, staff members who will have access to the data shall sign the Confidentiality Agreement listed in Appendix A, and signed copies shall be provided to CTUIR in a timely fashion.

6. Risks and Benefits

- Potential risks: The project volunteers should experience no discomfort from the use of the air monitoring equipment or urine tests. Loss of confidentiality is a potential risk, but medical data will be coded to maintain confidentiality and all materials and data will be stored in locked file cabinets and/or secured digital media.
- Potential benefits: This project will provide the community with a better understanding of how PAHs may be related to subsistence activities. This increased understanding regarding the risks of exposure to environmental PAHs can be used by Tribal members to develop culturally appropriate, practical risk reduction and outreach strategies. The approach that evolves from this project will also serve as a model to help other Tribal communities evaluate their PAH exposures and design risk reduction strategies that are protective of public health. Individual members may benefit through training in the proper use of the air sampling equipment for research and data collection procedures.
- Linkages: Because air quality data have the potential to affect an existing regulatory program, discussion about regulatory implications will occur with CTUIR staff before dissemination or use of the data. Because the health data have the potential to affect tribal health policies, the same provision will apply.

7. Communication of Research Results

The tribe will be kept fully apprised of research findings throughout the project. The methods of communication will be selected by CTUIR and OSU project leaders so as to provide the most effective dissemination. Potential avenues include: meetings with the grant Tribal Advisory Committee, meetings with the Health Commission, community meetings or forums, widely-distributed fact sheets, or articles in the local newspaper.

8. Publication or Presentation of Results

All publications and presentations developed using materials or data collected under this Agreement must be presented to the Director of the Department of Science and Engineering, CTUIR for review and approval prior to dissemination. This review process should take no longer than 30 days, unless an extension is mutually agreed upon by OSU and CTUIR leadership.

9. Compliance with Applicable Laws and Regulations

OSU and PNNL shall comply with all applicable federal laws and regulations protecting the privacy of citizens including the Family Educational Rights and Privacy Act (FERPA) and the Health Insurance Portability and Accountability Act (HIPAA).

10. Amendments and Alterations to this Agreement

CTUIR, PNNL, or OSU may amend this Agreement by mutual consent, in writing, at any time.

11. Termination of Services

In the event CTUIR, PNNL, or OSU terminates this Agreement, or any party ceases operation, or by 03/31/2015, OSU shall return to CTUIR all Material and Data collected in the course of this research. OSU shall certify in writing within five business days that all copies of the data stored on Program servers, backup servers, backup media, or other media including paper copies have been permanently erased or destroyed.

This Agreement contains all the terms and conditions agreed upon by the parties. No other understandings, oral or otherwise, regarding the subject matter of this Agreement shall be deemed to exist or to bind any of the parties hereto.

IN WITNESS WHEREOF, the parties have executed this Agreement:

STATE OF OREGON, Acting by and through the STATE BOARD OF HIGHER EDUCATION on behalf of OREGON STATE UNIVERSITY

____

Signature **Date**

____

Name (please print)

____

Title

____

Phone Number

CONFEDERATED TRIBES OF THE UMATILLA INDIAN RESERVATION

____

Signature **Date**

____

Name (please print)

____

Title

____

Phone Number

BATTELLE MEMORIAL INSTITUTE

____

Signature **Date**

____

Name (please print)

____

Title

____

Phone Number

Confidentiality Agreement

CTUIR-OSU Collaboration to Address Tribal Exposures to PAHs and Improve Community Health

Staff members with access to confidential client information are responsible for understanding the rules for use and disclosure of the information. This agreement verifies by signature that you agree to abide by the terms set out in the Material and Data Sharing Agreement, including the following provisions regarding the use and disclosure of client information:

1. Confidentiality of Client and Tribal Data

- Individually identifiable client data is confidential and is protected by various state and federal laws.
- Tribally identifiable data is confidential and is protected by various federal laws.
- Confidential client information includes all personal information (e.g., name, birth date, social security number, diagnosis, treatment) that may, in any manner, identify the individual.
- Confidential tribal information includes any information the CTUIR considers confidential (e.g., name, location, names of tribal leaders, or names of tribal representatives) that may, in any manner, identify the tribe.

2. Use of Client Data

- Client data may be used only for purposes directly described in agreements between the CTUIR and OSU.
- Any personal use of client information is strictly prohibited.
- Access to data is limited to personnel whose duties specifically require access to such data in the performance of their assigned duties.

3. Disclosure of Information

- Identified client information may not be disclosed to other individuals or agencies.
- Questions related to disclosure are to be directed to Anna Harding, Principal Investigator, Community Engagement Core.
- Any disclosure of information contrary to the provisions above is unauthorized and is subject to penalties identified in law.
- All elements remain in effect even after termination of your involvement or employment with the CTUIR-OSU Collaboration Program.

To be signed by all who have access to CTUIR material and data

I have read, understand my personal obligations, and am in agreement with the Material and Data Sharing Agreement and this Confidentiality Agreement.

____

Signature

____

Name (please print)

____

Title
